# Open to Contact? Increased State Openness Can Lead to Greater Interest in Contact With Diverse Groups

**DOI:** 10.1177/01461672211030125

**Published:** 2021-07-22

**Authors:** Victoria Hotchin, Keon West

**Affiliations:** 1 Goldsmiths, University of London, UK

**Keywords:** Openness, personality, prejudice, contact, intervention

## Abstract

Contact is a reliable method of prejudice reduction. However, individuals higher in prejudice are less interested in contact with diverse groups. This research investigates a novel method of encouraging interest in contact, particularly for those lower in the personality trait of Openness/Intellect, who tend to be higher in prejudice. Although long-term traits are relatively stable, momentary personality states show considerable within-person variation, and can be manipulated. In two experimental studies (total *N* = 687), we tested whether inducing higher state Openness would affect interest in contact. In Study 1, those lower in trait Openness/Intellect showed a positive indirect effect of condition on two outcome measures, via greater state Openness. In a larger sample with lower trait Openness/Intellect (Study 2), the indirect effect on the first outcome was replicated, regardless of disposition. The findings suggest that experiencing open states more frequently could encourage contact and lead to eventual reductions in prejudice.

The question of how to improve intergroup relations and reduce prejudice has received considerable research attention over recent decades. A key finding from the literature is that intergroup contact is one of the most effective known methods of prejudice reduction (Pettigrew & Tropp, 2006). Where favorable conditions exist, such as working toward a common goal and sharing equal status, positive contact between groups has been shown to reliably improve intergroup attitudes (Pettigrew et al., 2011).

However, there may be limits to the viability of contact in real-world situations. There have been a number of calls to assess the impact of multiple predictors on the effectiveness of contact for prejudice reduction, especially the role of individual differences (Hodson, 2009; Paolini et al., 2018). Encouragingly, recent research suggests that various forms of contact can be equally, if not more, effective for those who hold the most prejudiced attitudes at the outset, such as those higher in right-wing authoritarianism (Asbrock et al., 2013; Hodson, 2011; Hodson & Dhont, 2015; Kteily et al., 2019; West et al., 2017). However, even though intergroup contact may be effective for individuals higher in prejudice, they are among the least willing to actually engage in contact outside the laboratory (Hodson, 2011; Ron et al., 2017). This problem presents a serious barrier to the potential of contact to reduce prejudice.

This reluctance to engage in contact may be exacerbated by the fact that individuals higher in authoritarianism and prejudice tend to live in less diverse neighborhoods (Pettigrew, 2008), thereby further reducing their opportunities for contact. Those who do live in diverse areas may still avoid contact (Van Assche et al., 2019). Thus, it seems prudent to investigate ways of encouraging participation in contact, particularly for individuals who are high in prejudice or in other factors that make participation in contact less likely.

It is important that attempts to encourage interest in contact outside of the laboratory avoid some of the pitfalls of other intervention approaches. For example, research on the effects of diversity training and intervention programs in organizations indicates that they can “backfire” when individuals feel that they are being told what to do, and can entrench existing stereotypes and negative beliefs (Caleo & Heilman, 2019; Dobbin & Kalev, 2018). While laboratory-based contact interventions have been successful in reducing prejudice for those more prone to it, attempts to initiate contact opportunities in real-world settings could be more challenging. Although a meta-analysis of real-world contact interventions (Lemmer & Wagner, 2015) demonstrates their potential to work outside the laboratory, it is notable that the majority of studies included were conducted in schools and universities. This was also the case for studies in an additional meta-analysis of experimental contact interventions, where no studies on ethnic or racial contact included participants over age 25 (Paluck et al., 2019). There is, therefore, a relatively small number of studies that provide support for the success of contact interventions with non-student adults in naturalistic settings.

Given these potential difficulties, how might individuals higher in prejudice be encouraged to engage in intergroup contact? One approach is to begin by examining the personality factors that underlie the disposition toward prejudice. The personality trait of Openness to Experience (otherwise known as Openness/Intellect) reflects an interest in exploration and novelty, and is associated with curiosity, imagination, and creativity (DeYoung, 2014). Openness shows a reliable relationship with positive attitudes toward outgroups (Hotchin & West, 2018; Sibley & Duckitt, 2010) and is associated with greater likelihood of engaging in (Jackson & Poulsen, 2005) and benefiting from (Danckert et al., 2017) positive intergroup contact. Open individuals thrive when living in diverse cosmopolitan environments (Jokela et al., 2015).

In contrast, individuals lower in Openness have a preference for familiarity and stability, and seek to defend against threats to the status quo. As a result, they tend to be higher in authoritarian social attitudes and develop prejudice toward groups considered dissident or dangerous (Hotchin & West, 2018; Sibley & Duckitt, 2010). Resistance to contact with threatening outgroups is likely to result from this process (Hodson, 2011; Pettigrew, 2016).

Increasing interest in intergroup contact in such individuals would seem to be a difficult task. However, insights from personality psychology provide a framework for how such change might occur. Although traits such as Openness have traditionally been thought of as relatively stable, it is now recognized that they show developmental patterns across the life course (Roberts et al., 2006) and change in response to life events (Bleidorn et al., 2018), interventions (Roberts et al., 2017), and volitional goals (Hudson & Fraley, 2015). One theory of how such change occurs is via changes in personality states (Wrzus & Roberts, 2017).

Personality states are momentary expressions of a trait, reflected in an individual’s current thoughts, feelings, and behaviors. States are influenced by an individual’s tendencies, motivations, and the characteristics of the situations they experience (Fleeson, 2001; Fleeson & Jayawickreme, 2015). Traits correspond fairly closely to the average of a person’s state distribution. Therefore, one difference between individuals high and low in trait Openness/Intellect is that they will experience Open states more or less frequently. However, even an individual low in trait Openness/Intellect will express relatively high state Openness some of the time (Fleeson, 2001; Fleeson & Jayawickreme, 2015).

Recent research suggests that reflecting on nostalgic (van Tilburg et al., 2015), positive, and novel experiences (Hotchin & West, 2020) can increase state Openness. Enjoyment of exploration and novel experiences is characteristic of those higher in trait Openness/Intellect (McCrae & Costa, 1997), but the effects of the manipulation were found for both those lower and higher in trait Openness/Intellect (Hotchin & West, 2020). This suggests that momentarily re-experiencing past Open behavior through reflection causes an individual to feel more Open, regardless of whether they are high or low in Openness generally. This may be because recalling instances of successfully engaging in positive novel experiences increases confidence about opportunities for future exploration (Hotchin & West, 2020). An interesting question is whether feeling more Open has a follow-on effect with regard to attitudes toward contact. When individuals feel more Open, are they also more open to contact?

The implications of such a finding would be important for research on contact interventions. As well as providing a practical method of engaging individuals in contact when opportunities are available, it would also suggest a possible route toward a lasting change in attitudes. Theories of personality change suggest that it occurs via repeated changes in states (Wrzus & Roberts, 2017). If individuals experience more Open states repeatedly, they may integrate these changes into their self-concept implicitly, via habitual association, or via reflective processes, thereby seeing themselves as someone who is more “Open” (Wrzus & Roberts, 2017). In either case, if Open states lead to greater interest in contact (and opportunities for contact are available), this may eventually lead to a sustained decrease in prejudice.

## Study 1

In the present research, we tested whether increased state Openness would extend to an interest in contact with diverse groups. To induce greater state Openness, we used a manipulation found to be successful in previous research, whereby individuals reflect on positive novel experiences from their past (Hotchin & West, 2020). To assess interest in contact, we used two different measures. One was an established questionnaire assessing behavioral intentions regarding contact with diverse groups. The second was a novel measure of interest in contact included at the end of the study, which asked participants if they would be interested in taking part in a future study involving real-life meetings with diverse groups.

We expected that the experimental manipulation would induce higher state Openness in individuals both high and low in trait Openness/Intellect, as found in previous research (Hotchin & West, 2020). However, it was expected that experiencing greater state Openness may be more likely to affect interest in contact for participants relatively lower in trait Openness/Intellect. This is because higher trait Openness/Intellect is already strongly associated with interest in contact with diverse groups, so experiencing greater state Openness is less likely to have an additional influence on interest in contact for these individuals. Conversely, individuals low in trait Openness/Intellect are generally less interested in diverse contact, and also experience higher state Openness less frequently. Thus, experiencing greater state Openness is more likely to have an effect on the outcome variables for these individuals, should there be correspondence between attitudes at the trait and state level (i.e., if Openness/Intellect and interest in contact are correlated at both levels). We, therefore, tested a moderated mediation model, where the indirect effect of condition on interest in contact, via state Openness, was dependent on the level of trait Openness/Intellect.

We evaluate the following hypotheses, though note that these were not preregistered:

**Hypothesis 1 (H1):** There will be a main effect of event recall condition on state Openness. Participants who recall a positive novel event will have higher state Openness scores than those who recall an ordinary event.
**Hypothesis 2 (H2):** There will be a main effect of event recall condition on interest in contact. Participants who recall positive novel events will show greater willingness to engage in diverse contact, and greater interest in taking part in a future contact study, compared with those who recall an ordinary event.
**Hypothesis 3 (H3):** Increased state Openness will mediate the relationship between event recall condition and willingness to engage in diverse contact, and the relationship between condition and interest in taking part in the future contact study.
**Hypothesis 4 (H4):** Furthermore, the paths between state Openness and the contact outcome measures will be moderated by trait Openness/Intellect, such that the effects will be specific to/stronger for those lower in trait Openness/Intellect.

## Method

Materials, analysis code, and anonymized data for the present research can be accessed at https://osf.io/mkxg5/.

### Power Analysis

We based our power analysis on the results of previous research, which found effect sizes of *d* = 0.46 to *d* = 0.62 for state Openness following a positive and/or novel event recall manipulation (Hotchin & West, 2020). A power analysis indicated that a sample size of 152 would be required to detect an effect at the lower end of this range at an alpha level of .05, with 80% power. As the effect sizes for the other outcome measures were unknown, we increased the sample size to 200, allowing us to detect smaller effects of *d* = 0.4. We recruited 210 to allow for anticipated exclusions of 5% of the sample.

### Participants

Participants (*N* = 210) were recruited via the Prolific online platform (https://www.prolific.co/) and paid approximately £1.25 for their participation. Demographic filters were applied such that participants were U.K. nationals living in the United Kingdom who had English as a first language, were non-students, and aged 25 to 45. Following exclusions (reported below), the final sample of 180 was 51.1% female, and aged between 24 and 50 (*M* = 35.15, *SD* = 6.02). A total of 71.6% held a university degree. The majority (85%) of participants identified as White.

### Materials

Except where indicated, items were presented in a randomized order and answered using a sliding scale (1-5), where 1 = *Strongly disagree* and 5 = *Strongly agree.*

#### Trait openness/intellect

We assessed trait Openness/Intellect using a shortened version of the Big Five Aspect Scales (BFAS; DeYoung et al., 2007; Openness 20 items; Extraversion 20 items; Conscientiousness, Agreeableness, and Neuroticism 4 items each; randomized order). Although we did not intend to analyze the data for the latter three traits, these were collected to reduce the possibility of participants inferring the purpose of the study. Extraversion was included as an exploratory variable. Items were answered on a 5-point Likert-type scale where 1 = *Strongly disagree* and 5 = *Strongly agree*. The 10 items for Openness and Intellect were each averaged to form the aspect scores; the two aspect scores were then averaged to form the trait score. In addition to its role as a moderator in H4, trait Openness/Intellect was also included as a covariate in the analyses for H1 to H3, due to its expected association with the outcome variables and state Openness.

#### Event reflection task

Participants were randomly assigned to one of two conditions in which they were asked to recall an event from their past, generate four key words that described the event, and write a brief description of the event, including details of how it made them feel to recall it. Participants were not able to continue with the study until they had spent 3 min on this task, though they were able to take longer than this. Participants in the experimental condition recalled positive novel events, while those in the control condition recalled ordinary events. Wording for the task followed the protocol described by Sedikides et al. (2015) for the ordinary events. Participants were asked to “Please bring to mind an ordinary event in your life. Specifically, try to think of a past event that is ordinary. Bring this ordinary experience to mind. Immerse yourself in the ordinary experience. How does it make you feel?” Participants in the positive novel condition were asked to “Please bring to mind a positive novel event in your life. Specifically, try to think of a past event during which you experienced something new for the first time and enjoyed it,” as in Study 1 of Hotchin and West (2020).

#### Manipulation check

Following the event reflection task, participants were asked to rate their recalled event for novelty and positivity. The ratings were used to determine exclusions based on the criteria specified in Study 2 of Hotchin and West (2020). Ratings of nostalgia and sociality were also taken, but are not reported as they are not relevant to the hypotheses.^1^

#### State openness

We assessed state Openness using Saucier’s Big Five Mini-Markers (Saucier, 1994). We included all eight items for Openness (two reversed-coded) plus an additional item used in previous research (“curious”; Hotchin & West, 2020). Participants were asked to what extent they agreed with a statement beginning “Thinking about the event makes me feel . . .” followed by one of the items. The state Openness score is the mean of the nine items. We also included four items for Extraversion, and two each for Agreeableness, Neuroticism, and Conscientiousness, to increase variation in responses.

#### Willingness to engage in diverse contact

We assessed willingness to engage in diverse contact using 10 items from the 15-item Diversity of Contact subscale of the original Miville-Guzman Universality-Diversity Scale developed by Fuertes et al. (2000). We selected the items that referred to interest in future contact or activities, as these best represented behavioral intentions. Items include “I would like to join an organization that emphasizes getting to know people from different countries,” and “I am interested in going to exhibits featuring the work of artists from minority groups.”^2^

#### Interest in a future contact study

We measured interest in taking part in a future contact study at the end of the demographics and feedback section. Participants were asked, “Please let us know if you would like to participate in any of the following studies in the future,” followed by three checkboxes with the captions, “Life experiences and well-being (online),” “Real life meetings with diverse groups (offline),” “Self-development program (4 weeks via mobile app).” Participants could select more than one checkbox. Meetings with diverse groups was the item of interest, and this was coded as a binary variable indicating whether it had been checked or not.

### Procedure

We presented the study in a single session using the Qualtrics online platform. After giving consent, participants were randomly assigned to one of the two conditions. They first completed the BFAS, followed by the event reflection task. Afterward, participants completed the manipulation check, followed by the state personality items and the willingness to engage in diverse contact scale. The demographics and feedback page followed, with the interest in future contact study item included at the end of the page.

### Data Screening

Before analyzing the data, we screened participants for exclusion criteria, in keeping with that reported in Study 2 of Hotchin and West (2020). Three participants with zero variance (*s*^2^ < .05) on trait Openness/Intellect, state Openness, or willingness to engage in diverse contact before reverse-coding were removed, as was one participant who did not describe an event, leaving a total of 206. A further 26 participants were excluded due to event novelty ratings greater than 3.5 (on a scale of 1-5) for ordinary events (22 participants^3^) or novelty ratings below 2.5 for novel events (four participants). These cut-off points were intended to create a balance between ensuring the experimental conditions were sufficiently distinguished, without too great a loss of data.

Participants who were excluded were younger (*M* = 31.6, *SD* = 5.36) than those who were retained, *M* = 35.15, *SD* = 6.02; *t*(208) = 3.04, *p* = .003. They did not significantly differ on trait Openness/Intellect, excluded: *M* = 3.55, *SD* = 0.51, retained: *M* = 3.64, *SD* = 0.46; *t*(208) = .992, *p* = .322. Following these exclusions, the sample consisted of 180 participants.

It was also planned that if (following the above exclusions) outliers (±3 *SD*s from the mean) were present per condition for the primary dependent variables (DVs): state Openness and willingness to engage in diverse contact; or the event ratings of positivity and novelty, the analyses would be performed both with and without these outliers. Three such outliers were found: one with low positivity ratings in the novel condition, one with low novelty ratings in the novel condition, and one with a high novelty rating in the ordinary condition. The outliers are retained in the reported analyses. None of the results were substantively different when outliers were removed (see Supplemental Material).

## Results

Means and standard deviations per condition for the key variables are displayed in Table 1. Table 2 shows the percentage of participants in each condition who were interested in taking part in the future studies. Table 3 displays bivariate correlations. Trait Openness/Intellect (measured prior to the manipulation) did not significantly differ by condition, *t*(178) = −1.47, *p* = .143.

**Table 1.** Study 1: Means and Standard Deviations by Condition.

	Alpha	Ordinary (*N* = 80)		Novel (*N* = 100)	
		*M*	*SD*	*M*	*SD*
Age	—	35.51	6.26	34.86	5.83
Trait Openness/Intellect	.79	3.59	0.49	3.69	0.44
Trait Openness	.78	3.55	0.65	3.66	0.59
Trait Intellect	.79	3.63	0.53	3.72	0.59
State Openness	.80	3.01	0.71	3.45	0.53
Event positivity rating	—	3.70	1.18	4.77	0.36
Event novelty rating	—	1.45	0.56	4.72	0.41
Willingness to engage in diverse contact	.88	3.31	0.76	3.51	0.77

**Table 2.** Study 1: Percentages of Participants Within Each Condition Interested in Taking Part in the Future Study.

Future study option	Ordinary	Novel
Life experiences and well-being (online)	96.3%	97.0%
Real-life meetings with diverse groups (offline)	25.0%	39.0%
Self-development program (4 weeks via mobile app)	46.3%	60.0%
All studies selected	22.5%	34.0%

**Table 3.** Study 1: Bivariate Correlations (*N* = 180).

	Measure	1	2	3	4	5	6	7	8	9	10
1	Age	—									
2	Gender (0 = male)	−.024	—								
3	Trait Openness/Intellect	−.054	.154*	—							
4	Trait Openness	−.069	.211**	.805**	—						
5	Trait Intellect	−.013	.019	.754**	.216**	—					
6	State Openness	.125	−.081	.222**	.216**	.126	—				
7	Event positivity rating	.061	.054	.121	.104	.084	.548**	—			
8	Event novelty rating	−.039	.012	.093	.092	.051	.375**	.553**	—		
9	Willingness to engage in diverse contact	−.085	.170*	.358**	.424**	.118	.208**	.063	.136	—	
10	Interest in contact study (0 = not interested)	−.029	.135	.101	.095	.061	.110	.020	.121	.245**	—
11	Condition (0 = ordinary)	−.054	.003	.110	.093	.078	.336**	.539**	.959**	.125	.148*

* Correlation is significant at the .05 level (two-tailed). **Correlation is significant at the .01 level (two-tailed).

We also conducted tests of measurement invariance for the two continuous outcome variables (state Openness and willingness to engage in diverse contact), in case the constructs were perceived differently by participants depending on condition. We used Comparative Fit Index (ΔCFI) ≤ .01 as the criteria and tested metric, scalar, and residual invariance. Errors for the three negatively worded items were correlated. The initial configural model fit was not ideal, χ²(296) = 501.232, *p* < .001; CFI = .843, root-mean-square error of approximation (RMSEA) = .062, 90% confidence interval (CI) = [.053, .072]; however, as alpha reliabilities for the established scales were acceptable and our focus was on invariance across the conditions rather than the measurement model itself, we proceeded with the analysis. The difference between the metric and configural model (ΔCFI = .006) was acceptable, suggesting invariance. When comparing the scalar with the metric model, two parameters were required to be unconstrained (for the items “uncreative” and “curious”) after which partial invariance was demonstrated (ΔCFI = .004). Invariance between the residual and adjusted scalar model was also demonstrated (ΔCFI < .001). The results suggest that the same constructs were measured across the two conditions.

**H1:** Supporting our hypothesis and replicating previous research, participants in the positive novel event condition reported higher state Openness (*M* = 3.45, *SD* = 0.53) than those in the ordinary event condition, *M* = 3.01, *SD* = 0.71; *t*(143.84) = −4.61, *p* < .001, *d* = 0.71, 95% CI = [.41, 1.02], equal variances not assumed. This effect of condition held, *F*(1, 174) = 21.16, *p* < .001, η_p_^2^ = .11 when adjusting for trait Openness/Intellect, *F*(1, 174) = 9.35, *p* = .003, η_p_^2^ = .05; age, *F*(1, 174) = 5.15, *p* = .025, η_p_^2^ = .03; and gender, *F*(1, 174) = 2.58, *p* = .110, η_p_^2^ = .02.

Consistent with previous research, the effect was not moderated by trait Openness/Intellect, Δ*R*^2^ = .00, *F*(1, 173) = .189, *p* = .664.

**H2:** We next tested whether participants who recalled positive novel events showed greater interest in contact with diverse groups, compared with those who recalled ordinary events. Participants in the positive novel condition had higher scores (*M* = 3.51, *SD* = 0.77) on the willingness to engage in diverse contact measure than those in the ordinary condition (*M* = 3.31, *SD* = 0.76), but the difference was not statistically significant, *t*(178) = −1.69, *p* = .094, *d* = 0.26, 95% CI = [–.03, .56]. When adjusting for the influence of trait Openness/Intellect, *F*(1, 174) = 21.49, *p* < .001, η_p_^2^ = .11; age, *F*(1, 174) = .46, *p* = .497, η_p_^2^ = .00; and gender, *F*(1, 174) = 2.79, *p* = .097, η_p_^2^ = .02, the effect of condition was reduced further, *F*(1, 174) = 1.31, *p* = .254, η_p_^2^ = .01; however, the estimated marginal mean remained higher in the experimental condition (*M* = 3.47, *SE* = .07) compared with control (*M* = 3.34, *SE* = .08).

However, a logistic regression analysis indicated that event recall condition did significantly predict interest in taking part in the future contact study: χ^2^(1) = 4.01, *p* = .045, Nagelkerke *R*^2^ = .031. Participants who recalled positive novel events were 1.92 (*p* = .048, 95% CI = [1.01, 3.66]) times more likely to check the box expressing interest than participants in the ordinary recall condition (equivalent to *d* = 0.36). Expressed as percentages, 39% of participants in the positive novel condition were interested in taking part, compared with 25% of participants in the ordinary condition.

When including trait Openness/Intellect, age and gender in the analysis, the overall model was nonsignificant, χ^2^(4) = 8.51, *p* = .075, Nagelkerke *R*^2^ = .065, as was the effect of condition, odds ratio: 1.92, *p* = .053, 95% CI = [.993, 3.68], though the odds ratio was comparable and in the expected direction.

For comparison, we also tested whether participants in the positive novel condition showed greater interest in any of the other future studies (see Table 2 for details). Condition only significantly predicted interest in the diverse contact study, though it was close to significance for the self-development program (odds ratio: 1.74, *p* = .067, 95% CI = [.962, 3.16]).

Our hypothesis was, therefore, supported for one of the two outcome measures, though both were in the predicted direction.

**Figure 1.**
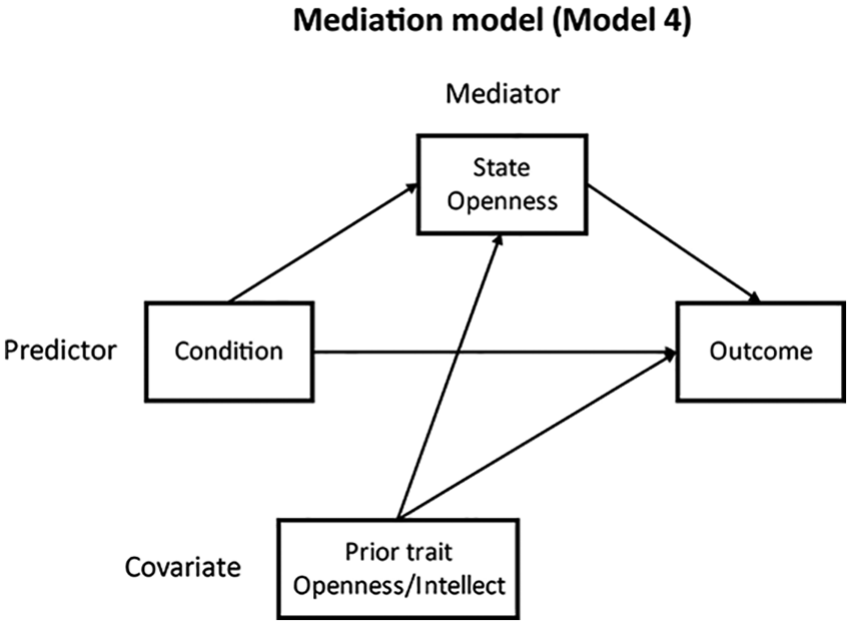
Mediation model structure.

**H3:** As an indirect effect can occur in the absence of a direct effect (Hayes, 2018), mediation models were tested for both outcome variables. Using Process v3.5 (Hayes, 2018), with 5,000 bootstrap samples, Model 4 was used to test whether there was evidence of an indirect path from event recall condition to willingness to engage in diverse contact, via increased state Openness. Trait Openness/Intellect was retained as a covariate due to its significant associations with the mediator and one outcome variable; age and gender were not. Figure 1 displays the model structure.

Event recall condition predicted state Openness (*b* = .41, *SE* = .093, *p* < .001, 95% CI = [.232, .594]), but state Openness did not significantly predict willingness to engage in diverse contact (*b* = .14, *SE* = .089, *p* = .116, 95% CI = [–.035, .316]). There was no indirect (*b* = .06, *SE* = .037, 95% CI = [–.015, .131]) or direct (*b* = .08, *SE* = .114, *p* = .503, 95% CI = [–.149, .302]) effect of condition.

We followed the same procedure using logistic regression to model the second outcome measure: interest in taking part in the future contact study. State Openness did not predict interest in taking part in the study (*b* = .19, *SE* = .276, *p* = .500, 95% CI = [–.355, .727]), and there was no significant direct (*b* = .54, *SE* = .349, *p* = .121, 95% CI = [–.142, 1.225]) or indirect (*b* = .08, *SE* = .118, 95% CI = [–.148, .324]) path from condition to this outcome measure.

The hypotheses were, therefore, not supported.

**Figure 2.**
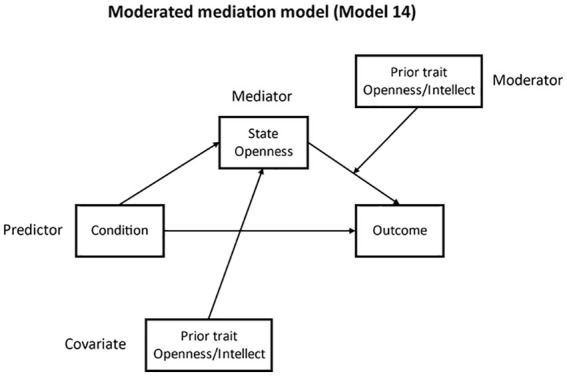
Moderated mediation model structure.

**H4:** We next tested a conditional mediation model (Model 14), where the path from state Openness to willingness to engage in diverse contact was moderated by trait Openness/Intellect, but not the path from condition to state Openness. This is because previous research did not find the effect of the experimental manipulation to be moderated by trait Openness/Intellect. As we expected the effect of condition to be mediated by state Openness, it was also not necessary to test the direct path for moderation. Trait Openness/Intellect was also included as a covariate for the mediator.^4^ Figure 2 displays the model structure.

We found evidence of moderated mediation (index of moderated mediation = −.12, *SE* = .069, 95% CI = [–.282, –.014]). For participants lower (–1 *SD* from the mean) in trait Openness/Intellect, there was a significant indirect path from event recall condition to willingness to engage in diverse contact, via state Openness (*b* = .11, *SE* = .052, 95% CI = [.025, .227]). This was not the case for participants with average (*b* = .06, *SE* = .037, 95% CI = [–.016, .133]) or high (+1 *SD* from mean; *b* = .00, *SE* = .045, 95% CI = [–.099, .079]) levels of trait Openness/Intellect. The model explained 16.7% of the variance in the outcome measure, *R*^2^ = .167, *F*(4, 175) = 8.74, *p* < .001.

We also found evidence of moderated mediation (index of moderated mediation = −.55, *SE* = .335, 95% CI = [–1.385, –.104]) when interest in the future contact study was the outcome variable. Consistent with the pattern reported above, for participants lower in trait Openness/Intellect, there was a significant indirect path from condition to interest in the future contact study, via state Openness (*b* = .39, *SE* = .215, 95% CI = [.075, .892]). This was not the case for participants with average (*b* = .14, *SE* = .127, 95% CI = [–.092, .401]) or high (+1 *SD* from mean; *b* = −.12, *SE* = .184, 95% CI = [–.554, .174]) levels of trait Openness/Intellect. Expressed as an odds ratio, participants lower in trait Openness/Intellect were 1.5 times (equivalent to *d* = 0.23) more likely to check the box indicating interest in the future contact study if they had experienced a 1-unit increase in state Openness after taking part in the positive novel condition. The model was significant (Model Lower Limit = 11.05, *df* = 4, *p* = .026, Nagelkerke *R*^2^ = .083).

Our hypothesis was, therefore, supported, indicating that for participants lower in trait Openness/Intellect, there was an indirect effect of condition on interest in contact across two outcome measures, via increased state Openness.

## Study 2

The results of Study 1 were encouraging, providing evidence that participants lower in trait Openness/Intellect were more open to engaging in future contact if they experienced greater state Openness following the experimental manipulation. This was the case for two different outcome measures: a self-report questionnaire and a more subtle measure of interest in a future contact study. To confirm the validity of these initial findings, we conducted a preregistered replication study with a much larger sample. In addition, as the effects found in Study 1 were specific to a subsection of the sample—those lower in trait Openness/Intellect—we aimed to recruit a sample with lower overall trait Openness/Intellect to have greater power to detect the effects.

We preregistered the same hypotheses, analyses, and criteria for data screening as Study 1 (https://osf.io/n2auf).

### Power Analysis

We aimed to recruit 550 participants. This sample size was within budgetary constraints and allowed us to detect an effect of *d* = 0.25 at 80% power with an alpha level of .05. For comparison, Study 1 found direct effects (without covariates) of condition on willingness to engage in diverse contact and the future contact study of *d* = 0.26 and *d* = 0.36, respectively.

### Participants

As in Study 1, participants (*N* = 552) were recruited via the Prolific platform, using the same demographic filters. We recruited participants in two batches of 275 each. For the second batch, we added an additional demographic filter that excluded participants with a university degree (undergraduate or postgraduate). In Study 1, participants with a university degree comprised 71.6% of the sample. The purpose of the filter was first, to better reflect the general population in the United Kingdom, where approximately 42% of working age adults have a university degree (Clegg, 2017), and second, to reduce the level of trait Openness/Intellect in the sample. This is because Openness tends to positively correlate with educational attainment (Lüdtke et al., 2011). This was important to do because the effects of the manipulation in Study 1 were specific to those below the mean in trait Openness/Intellect.

Following exclusions (reported below), the final sample of 507 were 57% female, and except for one participant aged 19, they were aged between 25 and 45 (*M* = 34.06, *SD* = 5.65). The majority (87.4%) of participants identified as White. Three participants were students, and 40% held a university degree. Participants were lower (*M* = 3.53, *SD* = 0.49) in trait Openness/Intellect than those in Study 1 (*M* = 3.64, *SD* = 0.46). This difference was statistically significant, *t*(685) = 2.70, *p* = .001, *d* = 0.23, 95% CI = [.06, .41], indicating the demographic filter was successful as a proxy for trait Openness/Intellect.

### Materials and Procedure

We used the same materials and procedure as in Study 1.^5^ However, because Study 2 was conducted while social restrictions related to the COVID-19 pandemic were in effect, we expected that participants may be more wary of social contact opportunities with strangers compared with prior to the pandemic, when Study 1 took place. We made two specific changes to the materials to attempt to mitigate this issue. The instructions for the willingness to engage in diverse contact scale were prefixed by the statement, “Looking ahead to a time when social distancing is no longer necessary”; and the future contact study wording was changed to “Online video meetings with diverse groups.” The latter change also had the advantage of increasing similarity between the format of the future study options presented, which were now all online-based studies.

### Data Screening

The same criteria used to screen the data in Study 1 were preregistered for Study 2. One participant with zero variance (*s*^2^ < .005) on state Openness was removed, as was one participant who did not describe an event, leaving a total of 550 participants.

A further 43 participants were excluded, due to event novelty ratings greater than 3.5 for ordinary events (37 participants) or novelty ratings below 2.5 for novel events (six participants).^6^ Participants who were excluded did not significantly differ by age, excluded: *M* = 33.02, *SD* = 5.44; retained: *M* = 34.06, *SD* = 5.65; *t*(550) = 1.18, *p* = .237; or trait Openness/Intellect, excluded: *M* = 3.47, *SD* = 0.39; retained: *M* = 3.53, *SD* = 0.49; *t*(56.87) = .965, *p* = .339. Following these exclusions, the sample consisted of 507 participants.

It was also preregistered that if (following the above exclusions) outliers (±3 *SD*s from the mean) were present per condition for the primary DVs: state Openness and willingness to engage in diverse contact; or the event ratings of positivity and novelty, the analyses would be performed with and without these outliers. A total of 14 such outliers were found: four with low positivity ratings and six with low novelty ratings in the novel condition; and four with high novelty ratings in the ordinary condition. The outliers are retained in the reported analyses. None of the results were substantively different when outliers were removed.

### Results

Means and standard deviations per condition are displayed in Table 4. Table 5 shows the percentage of participants in each condition who were interested in taking part in the future studies. Table 6 displays bivariate correlations. Trait Openness/Intellect (measured prior to the manipulation) did not significantly differ by condition, *t*(505) = .320, *p* = .749.

**Table 4.** Study 2: Means and Standard Deviations by Condition.

	Alpha	Ordinary (*N* = 239)		Novel (*N* = 268)	
		*M*	*SD*	*M*	*SD*
Age	—	34.23	5.59	33.91	5.70
Trait Openness/Intellect	.81	3.54	0.47	3.52	0.50
Trait Openness	.75	3.51	0.58	3.49	0.61
Trait Intellect	.82	3.56	0.62	3.56	0.64
State Openness	.80	2.88	0.70	3.37	0.53
Event positivity rating	—	3.93	1.23	4.75	0.41
Event novelty rating	—	1.44	0.64	4.75	0.40
Willingness to engage in diverse contact	.87	3.42	0.73	3.41	0.78

**Table 5.** Study 2: Percentages of Participants Within Each Condition Interested in Taking Part in the Future Study.

Future study option	Ordinary	Novel
Life experiences and well-being survey	95.0%	91.8%
Online video meetings with diverse groups (offline)	26.8%	28.4%
Self-development program (4 weeks via mobile app)	53.6%	55.6%
All studies selected	24.3%	26.5%

**Table 6.** Study 2: Bivariate Correlations (*N* = 507).

	Measure	1	2	3	4	5	6	7	8	9	10
1	Age	—									
2	Gender (0 = male)	−.073	—								
3	Trait Openness/Intellect	.038	.018	—							
4	Trait Openness	−.017	.107 *	.786**	—						
5	Trait Intellect	.075	−.073	.811**	.274**	—					
6	State Openness	.026	.019	.216**	.212**	.135**	—				
7	Event positivity rating	−.028	.057	.029	.027	.020	.483**	—			
8	Event novelty rating	−.015	.019	−.020	−.021	−.012	.404**	.439**	—		
9	Willingness to engage in diverse contact	−.133**	.179**	.405**	.439**	.214**	.227**	.078	−.014	—	
10	Interest in contact study (0 = not interested)	−.026	−.009	.075	.049	.071	.041	.027	−.010	.282**	—
11	Condition (0 = ordinary)	−.029	.020	−.014	−.019	−.004	.373**	.417**	.953**	−.009	.018

* Correlation is significant at the .05 level (two-tailed). **Correlation is significant at the .01 level (two-tailed).

We again tested for measurement invariance^7^ of state Openness and willingness to engage in diverse contact across conditions, using ΔCFI ≤ .01 as the criteria. Errors for the three negatively worded items were allowed to correlate. The initial configural model fit was similar to Study 1, χ²(296) = 781.31, *p* < .001; CFI = .854, RMSEA = .057, 90% CI = [.052, .062]. Differences between the metric and configural model (ΔCFI = .007), the scalar and metric model (ΔCFI = .009), and the residual and scalar model (ΔCFI = .009) were all below the threshold, demonstrating invariance and indicating that the same constructs were measured across the conditions.

**H1:** In keeping with Study 1 and prior research, participants in the positive novel event condition reported higher state Openness (*M* = 3.37, *SD* = 0.53) than those in the ordinary event condition, *M* = 2.87, *SD* = 0.70; *t*(441.64) = −8.90, *p* < .001, *d* = 0.81, 95% CI = [.62, .99], equal variances not assumed. This effect held, *F*(1, 504) = 87.99, *p* < .001, η_p_^2^ = .15, when adjusting for trait Openness/Intellect, *F*(1, 504) = 30.54, *p* < .001, η_p_^2^ = .06. In addition, and as expected, the effect was not moderated by trait Openness/Intellect, Δ*R*^2^ = .00, *F*(1, 503) = 2.55, *p* = .111.

**H2:** As in Study 1, there was no significant difference, *t*(505) = .21, *p* = .833, *d* = −0.02, 95% CI = [–.19, .16], between participants in the positive novel condition (*M* = 3.41, *SD* = 0.78) and the ordinary condition (*M* = 3.43, *SD* = 0.73) on willingness to engage in diverse contact. However, the scores across conditions were much more similar than in the previous study. Adjusting for the influence of trait Openness/Intellect, *F*(1, 504) = 98.97, *p* < .001, η_p_^2^ = .16, did not affect this result, *F*(1, 504) = .01, *p* = .929, η_p_^2^ = .00.

In contrast to Study 1, there was no effect of condition on interest in taking part in the future contact study: χ^2^(1) = .16, *p* = .691, Nagelkerke *R*^2^ = .00. Although more participants in the positive novel condition (28.4%) were interested in taking part compared with the ordinary condition (26.8%), the likelihood was not significantly different. Including trait Openness/Intellect in the analysis did not affect the result: χ^2^(2) = 3.07, *p* = .216, Nagelkerke *R*^2^ = .009, and trait Openness/Intellect was also not a significant predictor (odds ratio: 1.42, *p* = .089, 95% CI = [.948, 2.12]).

For comparison, we also tested whether participants in the positive novel condition showed significantly greater interest in any of the other future studies (see Table 5 for details); this was not found to be the case.^8^

**H3:** As noted previously, an indirect effect can occur in the absence of a direct effect (Hayes, 2018), therefore, mediation models were tested for both contact outcome variables, again using Process v3.5 (Hayes, 2018), with standard error estimators robust to heteroscedasticity (HC3) and 5,000 bootstrap samples.

Model 4 (see Figure 1) was used to test whether there was evidence of an indirect path from event recall condition to willingness to engage in diverse contact, via increased state Openness. Trait Openness/Intellect was included as a covariate. An indirect effect of condition on willingness to engage in diverse contact was found (*b* = .10, *SE* = .029, 95% CI = [.046, .161]). Event recall condition significantly predicted state Openness (*b* = .50, *SE* = .054, *p* < .001, 95% CI = [.393, .606]), and state Openness predicted willingness to engage in diverse contact (*b* = .20, *SE* = .055, *p* = .003, 95% CI = [.090, .305]). The direct effect of condition was not significant (*b* = −.10, *SE* = .066, *p* = .116, 95% CI = [–.235, .026]). The model explained 18.9% of the variance in the outcome measure, *R*^2^ = .189, *F*(3, 503) = 41.52, *p* < .001.

The same procedure was followed using logistic regression for the future contact study outcome. State Openness did not predict interest in taking part in the study (*b* = .07, *SE* = .168, *p* = .664, 95% CI = [–.256, .401]), and there was no significant direct (*b* = .05, *SE* = .217, *p* = .828, 95% CI = [–.377, .472]) or indirect (*b* = .04, *SE* = .085, 95% CI = [–.133, .202]) path from condition to this outcome measure.

The hypothesis was, therefore, supported for one of the two outcome measures.

**H4:** We next tested whether the path from state Openness to willingness to engage in diverse contact was moderated by trait Openness/Intellect (using Model 14; see Figure 2). Trait Openness/Intellect was also included as a covariate for the mediator. In contrast to Study 1, there was no evidence of moderated mediation (index of moderated mediation = −.05, *SE* = .048, 95% CI = [–.146, .043]). The indirect effect for participants lower in trait Openness/Intellect (*b* = .13, *SE* = .038, 95% CI = [.055, .206]) was not significantly different to the effect for participants average (*b* = .10, *SE* = .029, 95% CI = .049, .164) or higher (*b* = .08, *SE* = .037, 95% CI = [.009, .155]) in trait Openness/Intellect.

There was also no evidence of moderated mediation (index of moderated mediation = −.03, *SE* = .170, 95% CI = [–.370, .291]) when interest in the future contact study was the outcome variable. No indirect effects were found, regardless of level of trait Openness/Intellect.

## General Discussion

Across two studies, we investigated a novel method of increasing a person’s interest in intergroup contact. We found that when participants experienced greater state Openness following an experimental manipulation, they also expressed greater willingness to engage in contact with diverse groups. In Study 1, this effect was specific to those lower in trait Openness/Intellect, who typically show less interest in contact. In Study 2, we recruited a larger sample lower in trait Openness/Intellect overall, and found that the effect applied to all participants, regardless of level of trait Openness/Intellect.

We also used a novel, secondary measure of interest in contact, by asking participants if they were interested in taking part in a future contact study involving “real life meetings with diverse groups (offline).” In Study 1, there was a direct effect of condition on this measure, with participants in the positive novel condition showing greater interest in the study. In addition, we found that participants lower in trait Openness/Intellect who experienced greater state Openness after the manipulation were more likely to check the box indicating interest in the future contact study. In Study 2, which took place during the COVID-19 pandemic, we changed the future study description to “online video meetings with diverse groups,” but the direct and conditional indirect effects of condition did not replicate. We discuss possible reasons for the consistencies and discrepancies in our results below.

The personality trait of Openness/Intellect is an established predictor of both greater tolerance (Sibley & Duckitt, 2010) and greater engagement in contact with diverse groups (Jackson & Poulsen, 2005). Although both traits and social attitudes such as tolerance are considered relatively stable, how an individual thinks, feels, or acts at a particular moment is sensitive to situational factors as well as dispositional orientation. Individuals show considerable variation in the personality states they express, and even those lower in trait Openness/Intellect can express relatively higher states of Openness some of the time (Fleeson, 2001; Fleeson & Jayawickreme, 2015).

Following previous research (Hotchin & West, 2020), across two studies, we replicated the effect of an experimental manipulation on state Openness, finding that participants who reflected on positive novel experiences from their past (compared with ordinary events) felt more Open. Importantly, as in previous research (Hotchin & West, 2020), this effect occurred regardless of whether the participant was high or low in trait Openness/Intellect. That is, the manipulation increased state Openness independently of disposition.

It should be noted that while previous work on inducing personality states has tended to ask participants to enact trait-relevant behavior (e.g., Fleeson et al., 2002; McNiel & Fleeson, 2006), this manipulation is different in that it requests that participants reflect on instances of past trait-relevant behavior instead. However, personality states describe thoughts and feelings as well as outwardly manifested behavior, and Openness in particular is known to comprise largely cognitive content (Wilt & Revelle, 2015), which includes reflection and imagination. Therefore, the manipulation may be effective not only because of the recall of trait-relevant behavior, but because the act of deep reflection may itself be considered a manifestation of state Openness.

The novel contribution of our research was to assess whether Open states would affect attitudes toward contact. Given that trait Openness/Intellect is a significant predictor of interest in contact, we expected that individuals who already held positive attitudes about contact would maintain these, rather than show greater interest. We were more interested in whether individuals who are less Open—and, as a result, experience high Openness states less frequently—would change their attitudes when feeling more Open. We found that this was, indeed, the case. In Study 1, only individuals low in trait Openness/Intellect showed an indirect effect of the experimental manipulation on interest in contact, via state Openness. In Study 2, where trait Openness/Intellect was lower overall, the effect applied regardless of level of trait Openness/Intellect. Therefore, the moderation effect could be considered redundant where the sample is lower in trait Openness/Intellect.

This finding has important implications for our understanding of how prejudice develops and is maintained. Contact is well known to be a predictor of lower prejudice (Pettigrew & Tropp, 2006), and it is also established that individuals higher in Openness show greater interest in intergroup contact (Jackson & Poulsen, 2005), likely due to their general interest in variety and exploration (McCrae & Costa, 1997). An important driver of contact may, therefore, be Open states—which individuals higher in trait Openness/Intellect necessarily experience more frequently. The current research, as well as the previous research we replicated (Hotchin & West, 2018), indicates that state Openness is subject to situational influences. One route to prejudice reduction, therefore, may be via increased frequency of Open states.

Theory suggests that personality traits change as a result of repeated changes in the states that an individual experiences (Wrzus & Roberts, 2017). This leads to change either through habitual associations, where an individual maintains the repetition and it becomes part of their implicit self-concept, or the individual consciously reflects on their changed states and incorporates this into their explicit self-concept, such that it would be reflected on a trait questionnaire (Wrzus & Roberts, 2017). An important line of future research would be to see whether repeated changes in states also result in consistent and lasting changes in social attitudes, such as attitudes toward contact, at either the implicit or explicit level.

An important advantage of this approach to change is that it is indirect. Although laboratory research demonstrates that contact interventions are effective, particularly for those higher in prejudice (Hodson, 2011), there is less evidence of their success with non-student adults in real-world settings (Lemmer & Wagner, 2015; Paluck et al., 2019), where individuals higher in prejudice tend to show little interest in engaging in contact (Pettigrew, 2008). Furthermore, prejudice reduction interventions implemented in organizations, such as diversity programs, have been found to backfire due to participants being aware of the purpose of the intervention and, hence, unwilling or resistant to taking part (Caleo & Heilman, 2019; Dobbin & Kalev, 2018). This may be especially the case for those higher in prejudice, who are the target of such interventions (Hodson, 2011). These problems are avoided when the focus of change is Openness, with increased interest in contact a fortunate by-product.

A further strength of our research is that we applied a systematic approach to verify the conclusions of Study 1. We preregistered a replication study with a much larger sample size, and successfully recruited a sample with lower trait Openness/Intellect, as these were the participants who showed an effect of the manipulation in Study 1. Unfortunately, we were limited by the fact that the second study was conducted during the COVID-19 pandemic, while restrictions on social contact were still in place, though gradually easing. We expected these social conditions to have an impact on our contact outcome variables, and attempted to mitigate this by adjusting the wording. For the willingness to engage in diverse contact measure, we asked participants to look ahead to a time when social distancing was no longer required. This appeared to be effective, as the effects on this outcome variable were replicated.

Regarding the future contact study option, we adjusted this to be framed as an online study involving video meetings with diverse groups. Although the contact study was the least popular option for participants in both studies, and interest remained similar for participants in the ordinary condition, in Study 2, the level of interest was not elevated in the positive novel condition, as it had been in Study 1. One possible explanation for this is that participants had become very familiar with and possibly fatigued by online video meetings due to engaging in them more frequently during the pandemic. While “real life meetings with diverse groups” may sound appealing to someone experiencing greater state Openness and looking ahead to future novel opportunities, online video meetings may not elicit the same enthusiasm, especially if one is hoping to emerge from the pandemic restrictions soon.

### Limitations and Future Directions

One limitation of our research is that we relied on self-report measures of interest in contact. Although we used two different outcome measures, one of which was more subtle and may have elicited more spontaneous responses, both could still be subject to self-presentation bias. In addition, because no actual or virtual contact took place as part of the studies, we were not able to determine how a contact experience may play out when a person is experiencing greater state Openness.

Future research could directly involve participants in a contact scenario after the manipulation, for example, by using video conferencing to engage individuals from different groups in a cooperative exercise together. Assessing the effects of state changes on behavior in this way could have important implications for the implementation of contact interventions in organizations. For example, reminding participants of past positive novel experiences prior to recruitment or engagement in a contact opportunity could encourage greater uptake and interest. Ideally, the past novel experiences could be ones engaged in as part of the organization, for example, social activities and outings.

Our findings also imply that there are likely conditions that would have the opposite effect on state Openness, and thus decrease interest in contact (perhaps more so for those higher in trait Openness), for example, reminding participants of negative or threatening past experiences. Future research could investigate under what conditions individuals are less interested in contact, and the implications for contact interventions.

In addition, although individuals in our studies reported greater interest in engaging in future contact when feeling more Open after the manipulation, we do not know how long the effects persisted. While a short-term change could be helpful within a situation where contact is a current possibility, for example, when implementing contact interventions, more lasting changes are likely needed to create a sustained increase in contact in naturalistic settings, and subsequent reductions in prejudice. As mentioned, this may occur via the repetition of Open states over time. However, it would undoubtedly intersect with other barriers to contact that happen at the macro and meso levels, such as cultural and institutional norms, geographical dispersion, and group identities (Ron et al., 2017). Lasting change likely requires a multipronged approach considering influences at each of these levels.

Although increasing state Openness may seem a more viable approach than directly reducing prejudice in those who are most prone to it, there is also still a question of how to inspire interest in such a goal. Fortunately, as well as being associated with lower prejudice, Openness has a number of attractive associations with other outcomes. For example, individuals high in Openness adapt well to change and uncertainty, finding opportunities to learn and grow from adversity (Blackie et al., 2014). Furthermore, greater Openness is associated with cognitive health in older age (Franchow et al., 2013), possibly due to engagement in a greater variety of activities (Jackson et al., 2019). When individuals feel more Open, they also feel more like their authentic selves (Fleeson & Wilt, 2010). Therefore, increased Openness is a worthy goal in itself, aside from its effects on intergroup attitudes.

## Conclusion

The research presented represents a first step in understanding how changes in state Openness can affect attitudes toward contact, an important social concern. Replicating and extending previous research, we showed that when individuals feel more Open, they may also feel more open to contact. The results suggest a means by which to encourage participation in intergroup contact, particularly for those who may be the least interested to begin with.

## Supplemental Material

sj-docx-1-psp-10.1177_01461672211030125 – Supplemental material for Open to Contact? Increased State Openness Can Lead to Greater Interest in Contact With Diverse Groups Click here for additional data file. Supplemental material, sj-docx-1-psp-10.1177_01461672211030125 for Open to Contact? Increased State Openness Can Lead to Greater Interest in Contact With Diverse Groups by Victoria Hotchin and Keon West in Personality and Social Psychology Bulletin

sj-pdf-2-psp-10.1177_01461672211030125 – Supplemental material for Open to Contact? Increased State Openness Can Lead to Greater Interest in Contact With Diverse Groups Click here for additional data file. Supplemental material, sj-pdf-2-psp-10.1177_01461672211030125 for Open to Contact? Increased State Openness Can Lead to Greater Interest in Contact With Diverse Groups by Victoria Hotchin and Keon West in Personality and Social Psychology Bulletin

## Notes

1 For replication purposes, following the manipulation check, participants also completed a measure of the state functions of nostalgia originally used by Hepper et al. (2012) and reported in Study 1 of Hotchin and West (2020). The results (though consistent with previous research) are not reported in the present research as they are not relevant to the hypotheses.

2 For exploratory purposes, a feeling thermometer measure of prejudice developed by Asbrock et al. (2010) was included after the willingness to engage in diverse contact scale. The results are not reported as they are not relevant to the hypotheses of the present research.

3 This was a greater number of exclusions than we anticipated, based on previous research. It may be due to the wording in the study advertisement and information sheet (prior to the experimental task) stating participants would be asked to describe “an event” from their life rather than “events” (as in Hotchin & West, 2020). Some participants may have tried to second-guess the purpose of the study and provide a “good” event, even in the ordinary condition. As this number was larger than anticipated, the analyses were also run with these participants retained, and are reported in the Supplemental Material.

4 Model 14 incorporates the independent effect of the moderator on *Y* as well as the interaction effect.

5 As in Study 1, the state functions of nostalgia measure was also included immediately after the manipulation check, for purposes unrelated to the present study.

6 We changed the information sheet to refer to “events” rather than “an event,” which may have helped to reduce the proportion of exclusions compared with Study 1. However, as this is still a relatively high number of exclusions, the results with these 43 participants retained are presented in the Supplemental Material. The results are substantively the same, however, for Hypothesis 3, the indirect effect on willingness to engage in diverse contact was slightly weaker, and the direct effect of condition was significant and negative.

7 This analysis was not preregistered.

8 This analysis was not preregistered.
