# Supplementary material for: Open to Contact? Increased State Openness Can Lead to Greater Interest in Contact With Diverse Groups
Source: Pers Soc Psychol Bull. 2021 Jul 22;48(8):1177–90. doi: 10.1177/01461672211030125 (PMC9245154; doi:10.1177/01461672211030125)
Supplement: sj-docx-1-psp-10.1177_01461672211030125 – Supplemental material for Open to Contact? Increased State Openness Can Lead to Greater Interest in Contact With Diverse Groups [file sj-docx-1-psp-10.1177_01461672211030125.docx]

**Supplementary Materials**

**Study 1 results with 26 participants who failed the manipulation check for event ratings retained (*N* = 206):**

**H1:** Participants in the positive novel event condition reported higher state Openness (*M* = 3.45, *SD* = .54) than those in the ordinary event condition (*M* = 3.10, *SD* = .70); *t*(189.45) = -4.03, *p* < .001, *d* = .56 (95% CI = .29, .84), equal variances not assumed. The effect of condition held (*F* (1,200) = 13.90, *p* < .001, *η_p_^2^* = .07) when adjusting for trait Openness/Intellect (*F* (1,200) = 10.16, *p* = .002, *η_p_^2^* = .05), age (*F* (1,200) = 2.94, *p* = .088, *η_p_^2^* = .02) and gender (*F* (1,200) = 2.51, *p* = .115, *η_p_^2^* = .01).

Consistent with Studies 4 and 5, the effect was not moderated by trait Openness/Intellect (*ΔR^2^* = .00, *F* (1, 199) = .01, *p* = .931).

**H2:** Participants in the positive novel condition had higher scores (*M* = 3.51, *SD* = .77) on the willingness to engage in diverse contact measure than those in the ordinary condition (*M* = 3.39, *SD* = .75), but the difference was not statistically significant: *t*(204) = -1.16, *p* = .247, *d* = .16 (95% CI = -0.11 - 0.44). When adjusting for the influence of trait Openness/Intellect (*F* (1,200) = 26.37, *p* < .001, *η_p_^2^* = .12), age (*F* (1,200) = 1.39, *p* = .240, *η_p_^2^* = .01) and gender (*F* (1,200) = 2.19, *p* = .141, *η_p_^2^* = .01), the effect of condition was reduced to zero (*F* (1,200) = .47, *p* = .496, *η_p_^2^* = .00).

However, a logistic regression analysis indicated that event recall condition did significantly predict interest in taking part in the future contact study: *χ^2^*(1) = 4.50, *p* = .034, Nagelkerke *R^2^* = .030. Participants who recalled positive novel events were 1.88, *p* = .035 (95% CI = 1.04, 3.39) times more likely to check the box expressing interest than participants in the ordinary recall condition (equivalent to *d* = .35). Expressed as percentages, 40.4% of participants in the positive novel condition were interested in taking part, compared to 26.5% of participants in the ordinary condition.

When including trait Openness/Intellect, age and gender in the analysis, the effect of condition (but no other predictors) remained significant: odds ratio: 1.91, *p* = .034 (95% CI = 1.05, 3.46), but the overall model was not: *χ^2^*(4) = 6.45, *p* = .168, Nagelkerke *R^2^* = .043.

As such, the hypothesis was supported for one of the two outcome measures.

**H3:** An indirect effect of condition was found on willingness to engage in diverse contact (*b* = .06, *SE* = .028, 95% *CI* = .012, .123) Participants in the positive novel condition reported higher state Openness (*b* = .33, *SE* = .086, *p* < .001, 95% *CI* = .158, .498), and state Openness predicted greater willingness to engage in diverse contact (*b* = .20, *SE* = .080, *p* = .016, 95% *CI* = .037, .353). The direct path was not significant (*b* = .01, *SE* = .101, *p* = .946, 95% *CI* = -.194, .208). The model explained 15.8% of the variance in the outcome measure (*R^2^* = .158, *F*(3,202) = 12.62, *p* < .001).

The same procedure was followed using logistic regression to model the second outcome measure: interest in taking part in the future contact study. State Openness did not predict interest in taking part in the study (*b* = .26, *SE* = .255, *p* = .308, 95% *CI* = -.239, .758), and there was no significant direct (*b* = .53, *SE* = .311, *p* = .087, 95% *CI* = -.078, 1.142) or indirect (*b* = .09, *SE* = .087, 95% *CI* = -.069, .279) path from condition to this outcome measure. The model was not significant (Model LL = 6.18, *df* = 3, *p* = .103, Nagelkerke *R^2^* = .041).

The hypothesis was therefore supported for one of the two outcome measures.

**H4:** There was evidence of moderated mediation (index of moderated mediation = -.10, *SE* = .053, 95% *CI* = -.225, -.019) regarding the effect of condition on willingness to engage in diverse contact. For participants lower (-1SD from the mean) in trait Openness, there was a significant indirect path from event recall condition to willingness to engage in diverse contact, via state Openness (*b* = .11, *SE* = .043, 95% *CI* = .038, .206). This was also the case for participants with average (*b* = .06, *SE* = .028, 95% *CI* = -.013, .125) scores, but not for those with higher (+1 SD from mean; *b* = .02, *SE* = .031, 95% *CI* = -.049, .074) levels of trait Openness/Intellect. The model explained 17.9 % of the variance in the outcome measure (*R^2^* = .179, *F*(4,201) = 10.92, *p* < .001).

Evidence of moderated mediation (index of moderated mediation = -.37, *SE* = .230, 95% *CI* = -.948, -.053) was also found when interest in the future contact study was the outcome variable. Consistent with the pattern reported above, for participants lower in trait Openness, there was a significant indirect path from condition to interest in the future contact study, via state Openness (*b* = .29, *SE* = .159, 95% *CI* = .057, .672). This was not the case for participants with average (*b* = .12, *SE* = .094, 95% *CI* = -.037, .330) or high (+1 SD from mean; *b* = -.05, *SE* = .123, 95% *CI* = -.330, .168) levels of trait Openness/Intellect. Expressed as an odds ratio, participants lower in trait Openness/Intellect were 1.34 times (equivalent to *d* = .16) more likely to check the box indicating interest in the future contact study if they had experienced a 1-unit increase in state Openness after taking part in the positive novel condition. The overall model was significant (Model LL = 10.83, *df* = 4, *p* = .029, Nagelkerke *R^2^* = .071).

As such, the hypothesis was supported for both outcome measures.

**Study 2 results with 43 participants who failed the manipulation check for event ratings retained (*N* = 550):**

**H1:** In keeping with Study 1, participants in the positive novel event condition reported higher state Openness (*M* = 3.36, *SD* = 0.53) than those in the ordinary event condition (*M* = 2.94, *SD* = 0.70); *t*(510.91) = -7.91, *p* < .001, *d* = 0.67 (95% CI = .50, .85), equal variances not assumed. This effect held (*F* (1,547) = 68.61, *p* < .001, *η_p_^2^* = .11) when adjusting for trait Openness/Intellect (*F* (1,547) = 34.71, *p* < .001, *η_p_^2^* = .06). Additionally, the effect was not moderated by trait Openness (*ΔR^2^* = .00, *F* (1, 546) = 1.91, *p* = .168).

**H2:** In contrast to Study 1, there was no significant difference *t*(548) = .84, *p* = .401, *d* = -0.07 (95% CI = -.24, .10) between participants in the positive novel condition (*M* = 3.41, *SD* = 0.78) compared to the ordinary condition (*M* = 3.46, *SD* = 0.72) on willingness to engage in diverse contact. Adjusting for the influence of trait Openness/Intellect (*F* (1,547) = 97.50, *p* < .001, *η_p_^2^* = .15) did not affect this result (*F* (1,547) = .44, *p* = .507, *η_p_^2^* = .00).

There was also no effect of condition on interest in taking part in the future contact study: *χ^2^*(1) = .00, *p* = .957, Nagelkerke *R^2^* = .00. Although more participants in the positive novel condition (28.8%) were interested in taking part compared to the ordinary condition (28.8%), the likelihood was not significantly different. Including trait Openness in the analysis did not affect the result: *χ^2^*(2) = 2.83, *p* = .243, Nagelkerke *R^2^* = .007 and trait Openness was also not a significant predictor (odds ratio: 1.39, *p* = .094 (95% CI = .945, 2.05)).

As such, our hypotheses were not supported.

For comparison, we also tested whether participants in the positive novel condition showed greater interest in any of the other future studies (see Table 5 for details); this was not found to be the case.

**H3:** Model 4 (see Figure 1) was used to test whether there was evidence of an indirect path from event recall condition to willingness to engage in diverse contact, via increased state Openness. Trait Openness was included as a covariate.

An indirect effect of condition was found on willingness to engage in diverse contact (*b* = .09, *SE* = .024, 95% *CI* = .041, .136). Event recall condition significantly predicted state Openness (*b* = .43, *SE* = .052, *p* < .001, 95% *CI* = .326, .529), and state Openness predicted willingness to engage in diverse contact (*b* = .20, *SE* = .052, *p* < .001, 95% *CI* = .010, .304). The direct effect of condition was significant, and negative (*b* = -.13, *SE* = .062, *p* = .045, 95% *CI* = -.248, -.003). The model explained 17.9% of the variance in the outcome measure (*R2* = .179, *F*(3,546) = 41.16, *p* < .001).

The same procedure was followed using logistic regression to model interest in taking part in the future contact study. State Openness did not predict interest in taking part in the study (*b* = .07, *SE* = .158, *p* = .641, 95% *CI* = -.234, .380), and there was no significant direct (*b* = -.01, *SE* = .200, *p* = .944, 95% *CI* = -.407, .379) or indirect (*b* = .03, *SE* = .068, 95% *CI* = -.105, .165) path from condition to this outcome measure.

The hypothesis was therefore supported for one of the two outcome measures.

**H4:** We next tested whether the path from state Openness to willingness to engage in diverse contact was moderated by trait Openness (using Model 14). Trait Openness/Intellect was also included as a covariate for the mediator. In contrast to Study 1, there was no evidence of moderated mediation (index of moderated mediation = -.06, *SE* = .040, 95% *CI* = -.138, .023). The indirect effect for participants lower in trait Openness (*b* = .12, *SE* = .033, 95% *CI* = .054, .186) was not significantly different to the effect for participants average (*b* = .09, *SE* = .024, 95% *CI* = .045, .140) or higher (*b* = .06, *SE* = .029, 95% *CI* = .008, .122) in trait Openness.

There was also no evidence of moderated mediation (index of moderated mediation = -.04, *SE* = .139, 95% *CI* = -.329, .220) when interest in the future contact study was the outcome variable. No indirect effects were found, regardless of level of trait Openness/Intellect.

As such, our hypotheses were not supported.
