# Supplementary material for: Open to Contact? Increased State Openness Can Lead to Greater Interest in Contact With Diverse Groups
Source: Pers Soc Psychol Bull. 2021 Jul 22;48(8):1177–90. doi: 10.1177/01461672211030125 (PMC9245154; doi:10.1177/01461672211030125)
Supplement: sj-pdf-2-psp-10.1177_01461672211030125 – Supplemental material for Open to Contact? Increased State Openness Can Lead to Greater Interest in Contact With Diverse Groups [file sj-pdf-2-psp-10.1177_01461672211030125.pdf]

Materials

Trait Openness/Intellect

Instructions:

Here are a number of characteristics that may or may not describe you. For example, do you agree that you seldom feel blue, compared to most other people? Please select the option that best indicates the extent to which you agree or disagree with each statement listed below. Be as honest as possible, but rely on your initial feeling and do not think too much about each item.

[Strongly disagree] [Somewhat disagree] [Neither agree nor disagree] [Somewhat agree] [Strongly agree]

Presented in a randomised order: all 20 items from the BFAS (DeYoung et al., 2007) for Openness/Intellect and Extraversion, plus four items each for Conscientiousness, Agreeableness and Neuroticism.

Example items:

Enjoy the beauty of nature. [*Openness/Intellect*]

Make friends easily. [*Extraversion*]

Citation: DeYoung, C. G., Quilty, L. C., & Peterson, J. B. (2007). Between facets and domains: 10 Aspects of the Big Five. *Journal of Personality and Social Psychology, 93,* 880-896.

Event reflection task (control condition)

Instructions replicate those described for the control condition in Appendix B2 of Sedikides et al. (2015), and implemented in Study 1 and 2 of Hotchin and West (2021).

Citations:

Hotchin, V., & West, K. (2021). Reflecting on nostalgic, positive, and novel experiences increases state Openness. *Journal of Personality*, 89(2), 258-275.

Sedikides, C., Wildschut, T., Routledge, C., Arndt, J., Hepper, E. G., & Zhou, X. (2015). To nostalgize: Mixing memory with affect and desire. *Advances in Experimental Social Psychology* (1st ed., Vol. 51). Elsevier Inc. https://doi.org/10.1016/bs.aesp.2014.10.001

Event reflection task (experimental condition)

Instructions replicate those used for the positive novel condition in Study 1 of Hotchin and West (2021).

Citation: Hotchin, V., & West, K. (2021). Reflecting on nostalgic, positive, and novel experiences increases state Openness. *Journal of Personality*, 89(2), 258-275.

Manipulation check

Instructions:

Please indicate your agreement or disagreement with each of the following statements by moving the sliding scales to the left (disagree) or right (agree).

[Strongly disagree – Strongly agree]

Two items from the event characteristics measure used in Hotchin and West (2021):

The event I described was a positive experience

The event I described was a new experience for me

Citation: Hotchin, V., & West, K. (2021). Reflecting on nostalgic, positive, and novel experiences increases state Openness. *Journal of Personality*, 89(2), 258-275.

<u>State personality</u>

Instructions:

The following statements refer to how you feel after thinking about the event. Please indicate your agreement or disagreement with each statement by moving the sliding scales to the left (disagree) or right (agree).

Thinking about this event makes me feel...

[Strongly disagree – Strongly agree]

Presented in a randomised order: all eight Openness items from the Mini-Markers (Saucier, 1994) plus "curious"; four Extraversion items, and two items each for Conscientiousness, Agreeableness and Neuroticism.

Example items:

Imaginative *[Openness]*

Energetic *[Extraversion]*

Citation: Saucier, G. (1994). Mini-Markers: A Brief Version of Goldberg's Unipolar Big-Five Markers. *Journal of Personality Assessment*, 63(3), 506–516.

<u>Willingness to engage in diverse contact</u>

Instructions:

[Study 1]

Please indicate how descriptive each statement is of you by selecting the option corresponding to your response. This is not a test, so there are neither right nor wrong, good nor bad answers. All responses are anonymous and confidential.

[Study 2]

Looking ahead to a time when social distancing is no longer necessary, please indicate how descriptive each statement is of you by selecting the option corresponding to your response. This is not a test, so there are neither right nor wrong, good nor bad answers. All responses are anonymous and confidential.

[Strongly disagree – Strongly agree]

Presented in the following order: ten items from the Diversity of Contact subscale of the M-GUDS (Fuertes et al., 2000)

Items: 1, 2, 4, 7, 9, 13, 15, 16[1], 8, 30

Example items:

1) I would like to join an organization that emphasizes getting to know people from different countries.

[1] Item wording changed from 'United States' in the original questionnaire to 'UK'.

8) I am interested in going to exhibits featuring the work of artists from minority groups.

Citation: Fuertes, J. N., Miville, M. L., Mohr, J. J., Sedlacek, W. E., & Gretchen, D. (2000). Factor structure and short form of the Miville-Guzman Universality-Diversity Scale. *Measurement and Evaluation in Counseling and Development.*

Interest in future contact study

Instructions:

Please let us know if you would like to participate in any of the following studies in the future:

(More than one option can be selected)

[Study 1]

- ☐ Life experiences and well-being (online)
- ☐ Real life meetings with diverse groups (offline)
- ☐ Self-development program (4 weeks via mobile app)

[Study 2]

- ☐ Life experiences and well-being survey
- ☐ Online video meetings with diverse groups
- ☐ Self-development program (4 weeks via mobile app)
